# Flexible endoscopic micro-optical coherence tomography for three-dimensional imaging of the arterial microstructure

**DOI:** 10.1038/s41598-020-65742-2

**Published:** 2020-06-08

**Authors:** Junyoung Kim, Sunwon Kim, Joon Woo Song, Hyun Jung Kim, Min Woo Lee, Jeongmoo Han, Jin Won Kim, Hongki Yoo

**Affiliations:** 1 Mechanical Engineering Research Institute, KAIST, Daejeon, 34141 Republic of Korea; 2 Department of Cardiology, Korea University Ansan Hospital, Ansan, 15355 Republic of Korea; 3 Multimodal Imaging and Theragnostic Laboratory, Cardiovascular Center, Korea University Guro Hospital, Seoul, 08308 Republic of Korea; 4 Center for Robotics Research, Korea Institute of Science and Technology, Seoul, 02792 Republic of Korea; 5 Department of Mechanical Engineering, KAIST, Daejeon, 34141 Republic of Korea

**Keywords:** Imaging and sensing, Optical imaging, Interventional cardiology

## Abstract

Micro-optical coherence tomography (µOCT) is a novel imaging approach enabling visualization of the microstructures of biological tissues at a cellular or sub-cellular level. However, it has been challenging to develop a miniaturized flexible endoscopic µOCT probe allowing helical luminal scanning. In this study, we built a flexible endoscopic µOCT probe with an outer diameter of 1.2 mm, which acquires three-dimensional images of the arterial microstructures via helical scanning with an axial and lateral resolutions of 1.83 µm and 3.38 µm in air, respectively. Furthermore, the depth of focus of the µOCT imaging probe was extended two-fold using a binary phase spatial filter. We demonstrated that the present endoscopic µOCT could image cellular level features of a rabbit artery with high-risk atheroma and a bioresorbable scaffold-implanted swine coronary artery. This highly-translatable endoscopic µOCT will be a useful tool for investigating coronary artery disease and stent biology.

## Introduction

Optical coherence tomography (OCT) is an optical imaging technique that provides tomographic images of biological samples^1^. Owing to its high spatial resolution, fast imaging speed, and high sensitivity, OCT is currently widely used in diagnosing diseases and guiding treatment strategies in various medical fields, such as ophthalmology, oncology, and cardiology^2–7^. In particular, intravascular OCT has become a mainstay imaging modality for coronary artery disease, offering high-resolution imaging of luminal microstructures such as atherosclerotic plaques, intraluminal thrombus, bifurcation anatomy, and coronary stents^8–11^. Although OCT provides highly detailed images (spatial resolution of 10 to 30 μm)^12–14^, it is as yet insufficient for visualizing cellular- or subcellular-level structures, which may be essential for studying the progression and regression of the disease. To address this unmet need, researchers have striven to develop an advanced OCT imaging system with a higher resolution^15–17^, i.e., a micro-OCT (µOCT) technique, which, albeit being a bulky benchtop platform, now allows a spatial resolution of up to 1 μm^18^. The enhanced spatial resolution of µOCT enabled visualization of cellular structures in various organs, such as the blood vessels, colon, and cornea^15,18,19^.

To achieve cellular-level resolution, a broadband light source and high-numerical-aperture imaging lens must be used^20^, however, the utilization of such apparatus leads to an increase in imaging probe diameter, limiting its broader clinical applications. Furthermore, the depth of focus (DOF) shortens in inverse proportion to the square of the lateral resolution gain and the DOF shortening means that the area of visualization is reduced. Our group has recently developed a miniaturized endoscopic µOCT system that provides images with an axial and lateral resolution of 2.49 µm and 2.59 µm, respectively^21,22^. Together with a small-diameter gradient-index (GRIN) lens, a binary phase spatial filter (BPSF) was incorporated into the system to overcome the trade-off between the resolution and DOF; consequently the DOF was extended two-fold. Although the system successfully visualized the arterial microstructure via linear scanning, its application as an intravascular imaging modality was still limited due to a lack of helical scanning capability. In this study, we report our experience with using a flexible low-profile endoscopic µOCT imaging probe and a dedicated optical rotary junction that allow full 360° helical scanning. This cutting-edge µOCT imaging modality provides high-resolution tomographic images with axial and lateral resolution of 1.83 µm and 3.38 µm, respectively, while maintaining the extended DOF. To demonstrate the feasibility of the present technique for intravascular imaging, we tested it using a healthy swine coronary artery, a bioresorbable vascular scaffold (BVS)-implanted swine coronary artery, and a rabbit artery with high-risk atherosclerotic plaques.

## Results

### Characterization of the endoscopic µOCT system

**Figure 1:**
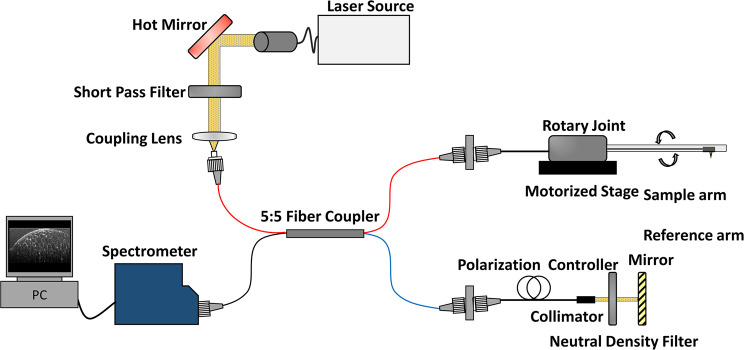
Schematic diagram of the developed endoscopic µOCT system for three-dimensional vascular imaging.

**Figure 2:**
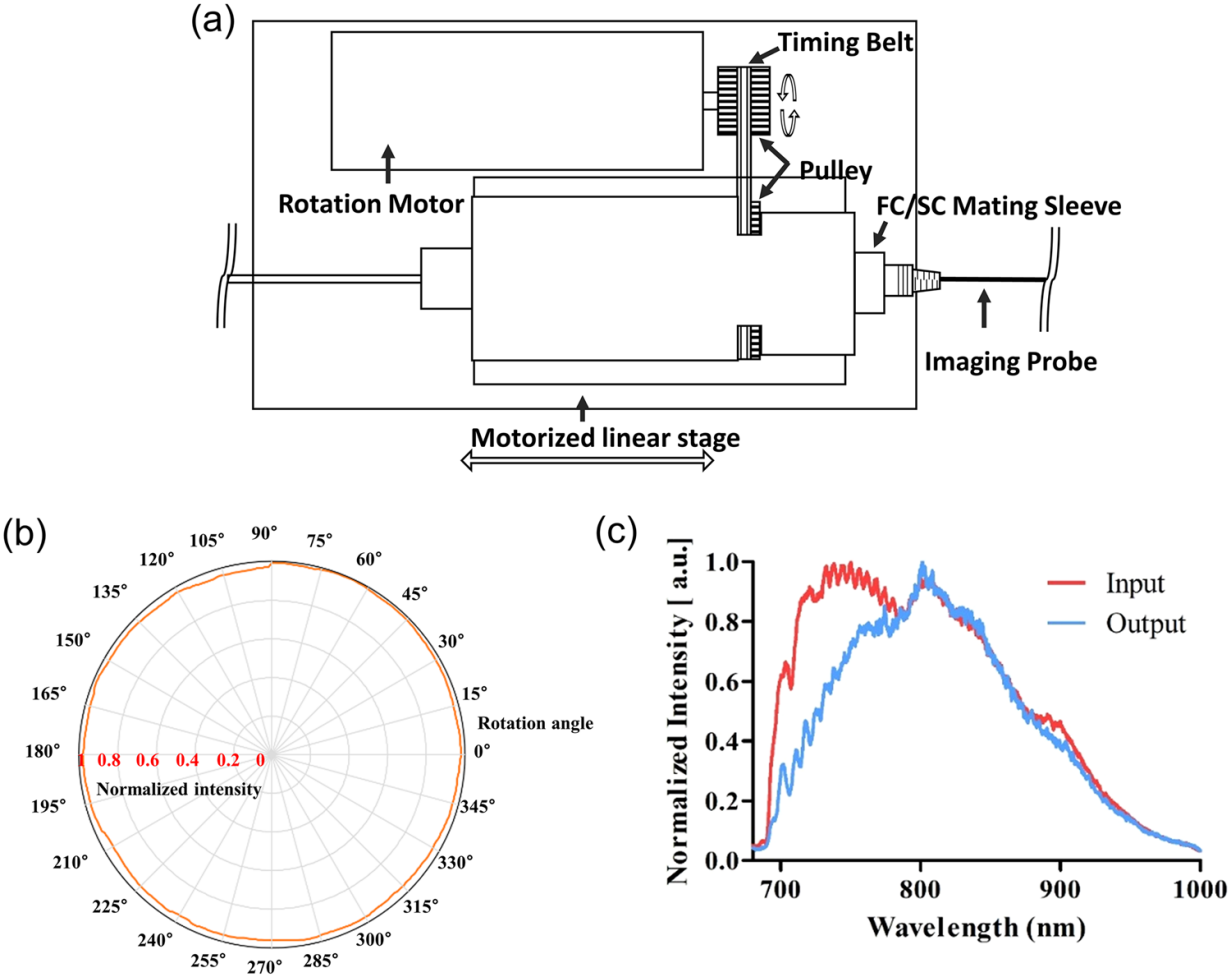
(**a**) Schematic diagram of the developed rotary joint and pullback stage for three-dimensional vascular imaging; (**b**) Experimental results of the normalized optical power throughput depending on the rotational angle of the rotary joint; (**c**) Experimental results of the spectral throughput of the rotary joint over the entire wavelength of the designed light source.

Figure 1 presents a schematic diagram of the endoscopic µOCT system. The system was built on the basis of a spectral-domain OCT using a supercontinuum laser (see the “Methods” section). The spectrometer determines the maximum a-line rate of the system at 40 kHz. The sensitivity of the system was 93.02 dB, which was measured using a mirror and neutral density filter. The scanning part was constructed using a laboratory-built rotary joint and motorized stage (see “Methods” section). A schematic diagram of the rotary joint developed for the three-dimensional vascular imaging is shown in Fig. 2a. The maximum rotational speed was 100 rps, and the measured throughput was 75.14% at center wavelength. The experimental transmission results depending on the rotational angle of the rotary joint are shown in Fig. 2b. The measured optical throughput variation upon rotation was within 6.2%. In addition, spectral measurements confirmed that all wavelengths in the ranges passed through the rotary joint, and there was no wavelength cut off as designed (Fig. 2c).

**Figure 3:**
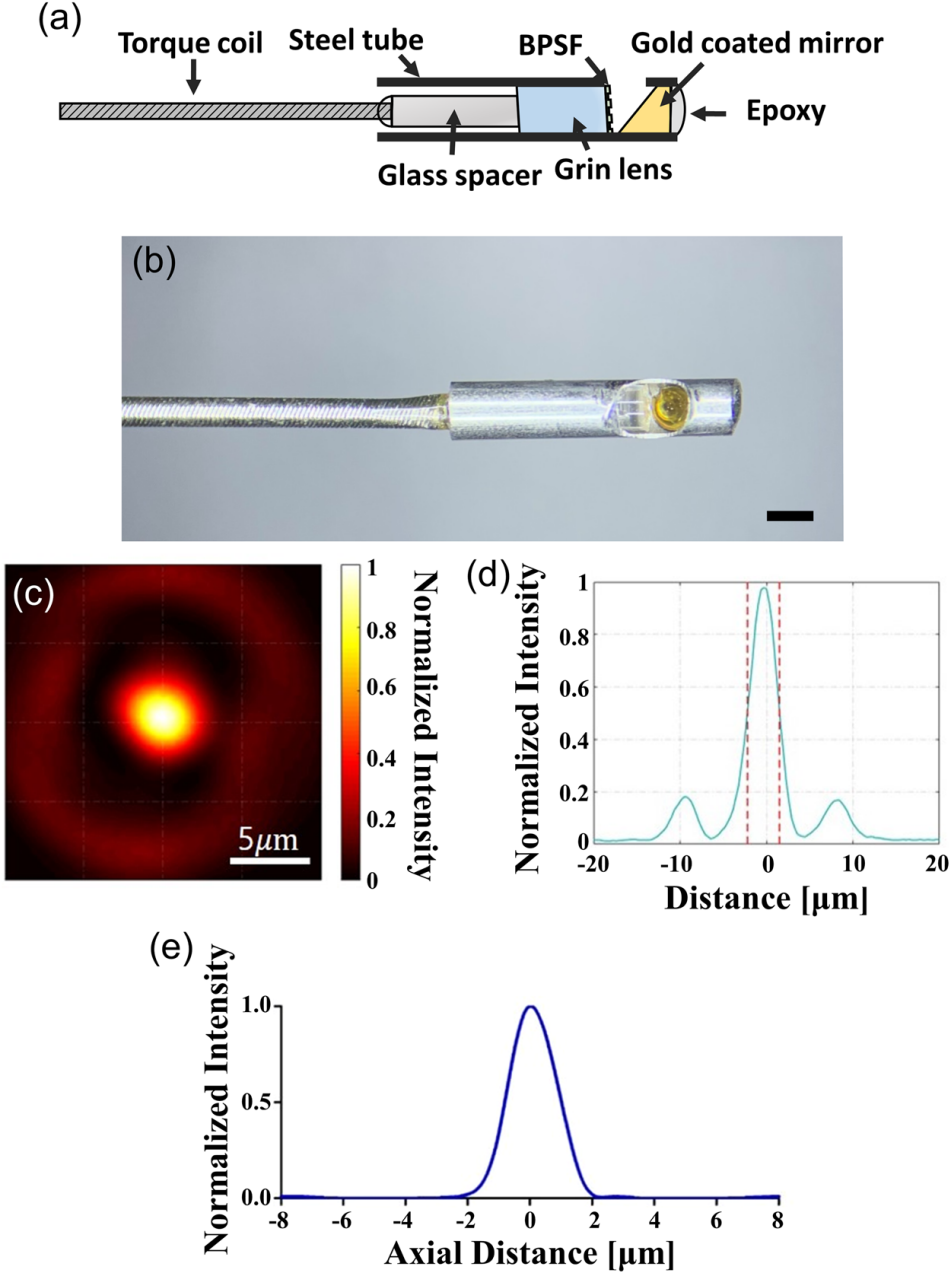
(**a**) Schematic diagram and (**b**) Photograph of the developed imaging probe. Scale bar, 1 mm; (**c**) Two-dimensional transverse PSF of the focused imaging system; (**d**) Cross-sectional profile of the PSF; (**e**) Normalized axial profile of a reflecting surface.

Figure 3a shows an imaging probe of the endoscopic µOCT system. The imaging probe was composed of 1 mm diameter optics and a binary phase spatial filter (BPSF) to increase the imaging range, which has a gain of 1.91. A photograph of the flexible imaging probe wrapped in a steel tube to protect it from impact and contamination is shown in Fig. 3b. The details of the fabrication methods are described in the “Methods” section. The length of the rigid portion of the imaging probe was 6.6 mm, and the outer diameter of the steel tube was 1.2 mm. Our flexible imaging catheter could provide images without non-uniform rotational distortion while the catheter shaft was being bent significantly (see Supplementary Materials and Fig. S1). The point spread function (PSF) of the imaging probe is shown in Fig. 3c. Figure 3d shows a representative cross-sectional profile of the transverse PSF. The lateral resolution, defined as full-width at half maximum (FWHM) of the intensity distribution, is 3.38 μm in the air. A normalized axial profile of a reflecting surface, shown in Fig. 3e, had a 1.83 μm axial resolution as calculated from the FWHM of the axial profile in the air.

### µOCT imaging of a healthy swine coronary artery

**Figure 4:**
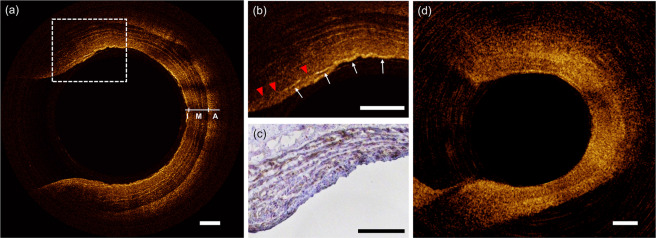
(**a**) µOCT image of a healthy swine coronary artery shows trilaminar vessel architecture of a muscular artery. I = intima; M = media; A = adventitia. (**b**) High magnification image at the bifurcation demonstrating partial loss (red arrowheads) of IEL (white arrows); (**c**) Corresponding immunohistological staining image for smooth muscle cells (α-SMA stain); (**d**) Image acquired using conventional OCT. Scale bars, 200 μm.

A freshly resected healthy swine coronary was imaged using a custom-made imaging chamber filled with phosphate-buffered saline (Supplementary Fig. S2). The fluorinated ethylene propylene-tubed µOCT imaging probe with an outer diameter of 1.65 mm was safely inserted into the artery. Figure 4a shows the representative cross-sectional image acquired using µOCT. Owing to the improved spatial resolution and increased DOF, the structure of the muscular arterial wall was well visualized as clearly distinguishable three layers, namely, tunica intima, tunica media and tunica adventitia (Fig. 4a). Furthermore, unlike conventional OCT showing the tunica media as a single ill-defined low-scattering layer, our µOCT technique fully characterized the laminated architecture of the medial layer consisting of multiple smooth muscle cell (SMC) sheets intermixed with elastic fiber (bright lines, Fig. 4a–c).

The internal elastic lamina (IEL) is a very thin elastic fiber layer separating the intima and media (Fig. 4b, white arrows) that is not readily identifiable with conventional OCT (Fig. 4d). In the process of atherosclerosis, the IEL serves as a restrictive barrier to molecular movement into the intima^23^. An injured IEL during angioplasty or stenting provides a gateway for immune cells and SMCs, consequently leading to excessive neointimal proliferation, i.e. restenosis. Furthermore, disruption or loss of the IEL contributes to the development of various vasculopathies such as coronary ectasia, aneurysm, and fibromuscular dysplasia^24,25^. With the present µOCT system, the IEL was clearly visualized as a highly scattering linear structure near the luminal surface (Fig. 4b, white arrows). A high index of refraction of elastin and its fibrous structure is thought to act as a robust scatterer of light^26^. We also detected fragmentation of the IEL (Fig. 4b, red arrowheads) at the coronary bifurcation, a preferred site for atherosclerosis where the endothelium is exposed to altered shear stress^27^. Previous experimental studies using surgically created bifurcation animal models reported a similar observation, i.e. IEL damage occurred at the periapical region of the bifurcation which experienced high shear stress^28,29^.

### µOCT imaging of coronary bioresorbable vascular scaffold (BVS) healing

**Figure 5:**
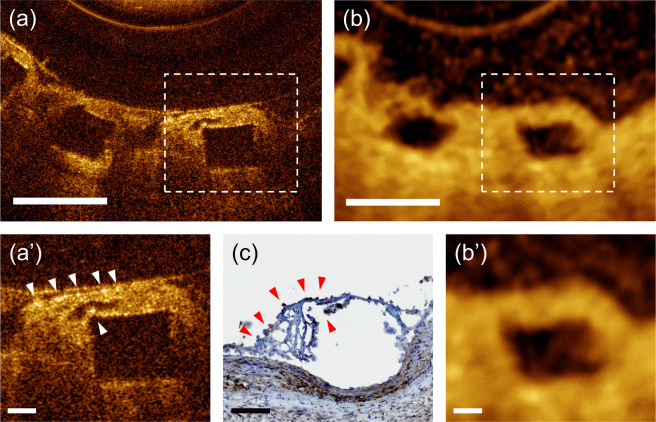
(**a**) Cross-sectional µOCT image of the swine coronary artery with a BVS implanted 7 days prior to imaging; (**b**) Corresponding conventional OCT image; (**a’**) A magnified µOCT image shows the infiltrated macrophages (bright spots, white arrowheads) around struts; (**b’**) Conventional OCT show neointimal tissue with relatively uniform signal intensity; (**c**) Corresponding anti-macrophage immunostaining findings corroborate the µOCT imaging findings (red arrowheads). Scale bars for **a** and **b**, 500 μm. Scale bars for **a’** and **b’**, 100 μm.

Coronary arterial healing following stent implantation is a natural reparative process involving a cascade of endothelial denudation, peri-strut blood clot formation, inflammatory reaction, and finally strut-coverage by newly formed intimal tissue, i.e. neointima^30^. Figure 5 shows the findings from a swine coronary artery with a BVS implanted 7 days ago. It is well known that stent-related arterial inflammation is mainly driven and regulated by macrophages^30,31^. After a neutrophil-mediated brisk early inflammatory response, monocytes infiltrate into the platelet-rich mural thrombus, forming aggregated macrophages clusters around struts^31^. As clearly shown in Fig. 5, the infiltrated macrophages, appeared as distinct bright spots in the µOCT (Fig. 5a and a), were present around the BVS struts. However, in conventional OCT, the BVS struts were surrounded by thick tissue with a relatively homogenous signal intensity (Fig. 5b and b). Peri-strut macrophage infiltration was verified in the corresponding histologic section stained with PM-2K anti-macrophage antibody (Fig. 5c).

**Figure 6:**
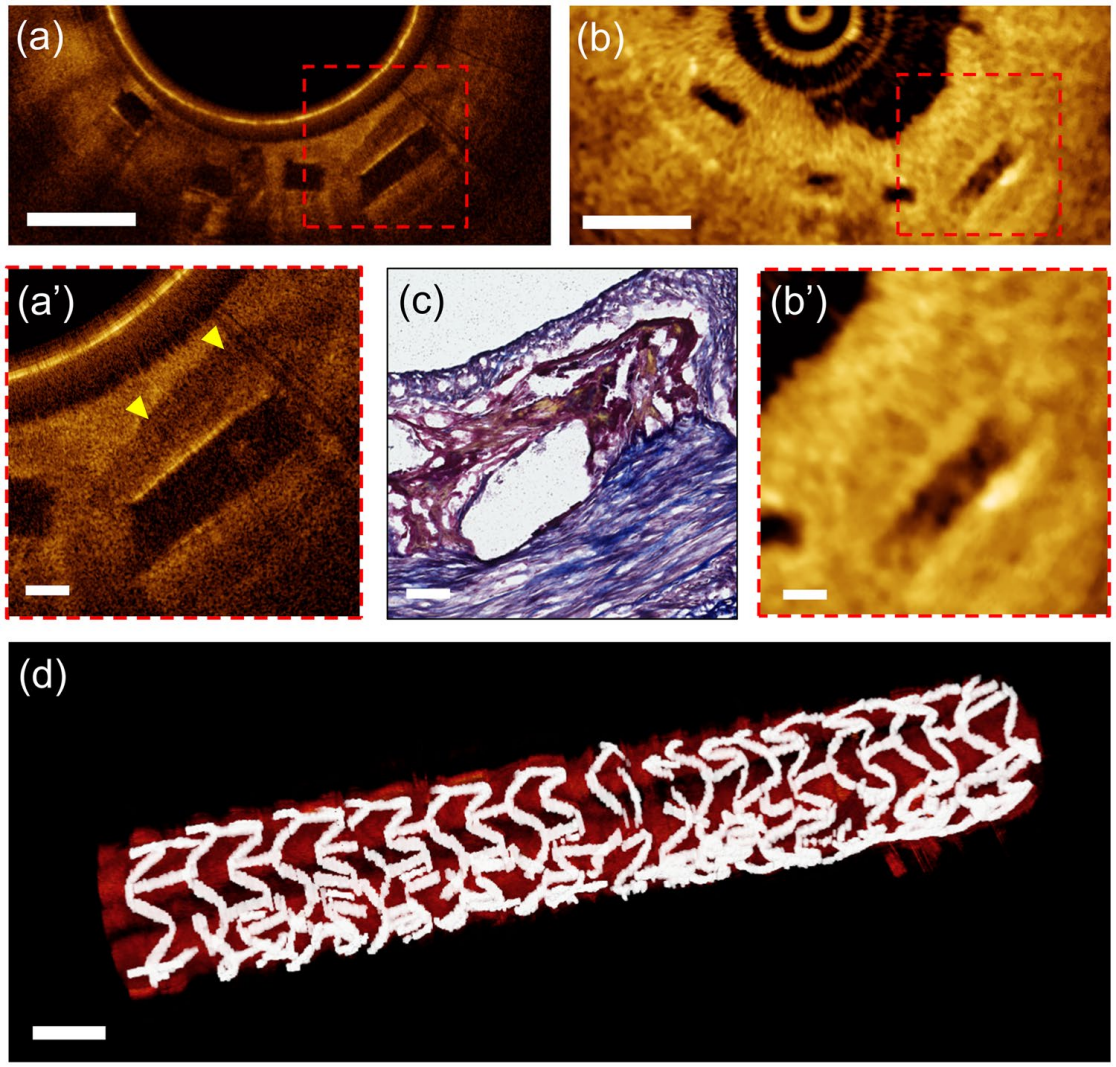
Imaging results of a swine coronary artery that received two overlapping BVSs (28 days post-implantation). (**a,a’**) µOCT image clearly demonstrating a peri-strut low-intensity area with well-defined margin (yellow arrowheads); (**b,b’)** Corresponding cross-section acquired using conventional OCT; (**c**) Histologic section shows excessive fibrin deposition around strut (Verhoeff elastic-Masson trichrome stain). (**d**) A three-dimensionally reconstructed image of BVSs-implanted swine coronary artery (28 days post-implantation) showing multiple fractured struts. Three-dimensional volume rendering was performed using OsiriX (The OsiriX Foundation, Geneva, Switzerland). Scale bars for **a**, **b**, and **d** 500 μm. Scale bars for **a’, b,’** and **c** 100 μm.

After intravascular stent implantation, the protruded strut disrupts the coronary laminar flow, creating a flow stagnation zone around the struts, which triggers platelet aggregation and fibrin deposition^32^. Fibrin, if deposited at a physiological level, is internalized and degraded by macrophages^30^. However, excessive fibrin deposition, typically observed as a peri-strut low intensity area on OCT, is a marker of maladaptive stent healing and a precursor of stent-related complication^33,34^. Also, experimental and clinical studies have clearly shown that the overlapping stent segments harbor more fibrin and inflammatory cell deposition than the non-overlapping sites^35^, and were associated with worse clinical outcomes^36^. Figure 6 presents a µOCT image obtained in an arterial segment stented with two overlapping BVSs. The entire µOCT imaging data is provided in Supplementary Movie 1. Using our µOCT technique, the peri-strut fibrin deposition (Fig. 6a,a and c) was more clearly visualized compared to that with conventional OCT (Fig. 6b and b). Moreover, even physiologic-level fibrin deposition could be observable with our µOCT imaging (Supplementary Fig. S3). The current imaging based on helical luminal scanning could readily provide a three-dimensional image of arterial structure of interest, facilitating an intuitive and comprehensive interpretation (Fig. 6d).

**Figure 7:**
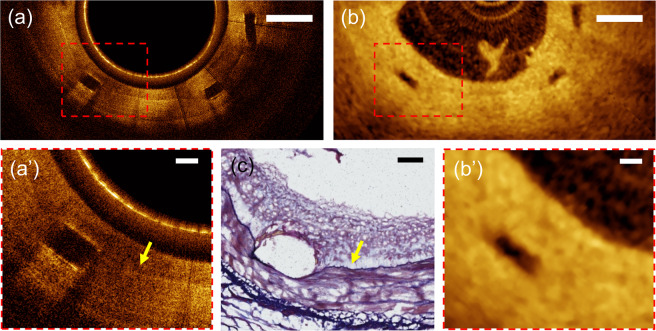
(**a**) μOCT image of a BVS-implanted swine coronary artery (28 days post-implantation) and (**b,b’**) corresponding cross-section of conventional OCT; (**a’**) A magnified μOCT image shows a thin highly-scattering IEL (arrow) that separates the medial layer and the strut-covering neointima; (**c)** Corresponding histologic section stained with Verhoeff elastic-Masson trichrome; Scale bars for a and b, 500 μm. Scale bars for a’, b’ and c, 100 μm.

The capability of µOCT to characterize the IEL was still effective in case of BVS-implanted artery covered with mature neointima (28 days post-implantation). Altered IEL geometry due to vascular scaffolding was clearly delineated with our µOCT (Fig. 7a and a’), while conventional OCT visualized a vague line connecting the contiguous struts (Fig. 7b and b’) in a limited area only. Fully expanded BVS struts compressed and stretched the IEL outward into the medial layer (Fig. 7a and c). The finding of correlative Verhoeff elastic-Masson trichrome (VMT) staining, which allows differentiation of elastin (black), corroborated accurately with the µOCT imaging findings (Fig. 7a and c) Neointimal thickness after stenting is an efficacy measure of individual stent and is currently calculated by drawing an arbitrary circular line interconnecting the stent struts^5^. However, as demonstrated in Fig. 7, the IEL geometry after stenting was not complete circular in shape and could be altered further in diseased arteries. Furthermore, coronary stenting inevitably causes hyperstretch vessel injury and resultant IEL rupture induces excessive neointimal proliferation, consequently leading to stent failure^37^. Such arterial response to severe vessel injury was still substantial despite the use of newer stents coated with antiproliferative drug^38^. The present higher-resolution approach is expected to provide better imaging guidance to optimize the stent implantation and to monitor the stent efficacy.

### µOCT imaging for detecting microcalcification in atherosclerotic plaques

**Figure 8:**
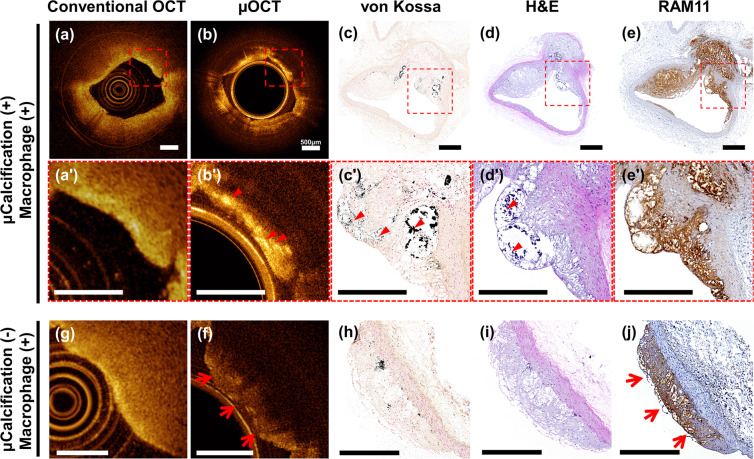
(**a – e’**) Comparison among conventional OCT, μOCT and histologic evaluation of a high-risk plaque with microcalcification; (**a,a’**) Conventional OCT images showing ill-defined signal-poor region; (**b,b’**) μOCT images showing multiple bright spots (red arrowheads); The red arrowheads indicate the corresponding microcalcification in μOCT (**b’**), von Kossa (**c’**) and H&E (**d’**), and RAM11 (**e’**) images; (**g–j**) Imaging findings from a plaque without surface microcalcification. Scale bars, 500 μm.

To investigate whether the current μOCT system can identify the microstructural feature that leads to atherosclerotic plaque destabilization, a rabbit model of atherosclerotic calcification was developed and imaged. In the cross-sections with bunch plaque formation, conventional OCT showed a poorly-delineated signal-poor lesion implying a high-risk plaque (Fig. 8a). Some punctate signal-rich structures near the luminal surface, albeit not clearly discernible, suggested the macrophages infiltration (Fig. 8a’). However, unlike the findings from conventional OCT, the abluminal area contained a multiple punctate or confluent bright spots with signal attenuation in μOCT (Fig. 8b and b’). To determine the origin of the spotty bright signals in μOCT image, we performed histologic validation using von Kossa staining for evaluating calcification (Fig. 8c and c’); haematoxylin-eosin (H&E) staining for morphological characteristics (Fig. 8d and d’); and RAM11 immunohistochemical staining for macrophage infiltration (Fig. 8e and e’). Histopathologic evaluation of the corresponding histologic section revealed that the lesion was macrophage-abundant plaque with microcalcification (Fig. 8c’–e’). The bright spots in the μOCT scans corresponded with the calcium-positive areas (black) in the von Kossa-stained histologic section (Fig. 8b’ and ç’), supporting that the current μOCT system could specifically detect the plaque microcalcification. Conversely, bright spots were not noted in the plaques without surface microcalcification (Fig. 8g-j).

Intimal microcalcification, i.e. calcium deposits approximately 5–60 μm in diameter, is currently considered a marker of plaque vulnerability because microcalcification amplifies the local stress on the fibrous cap and increase the risk of plaque rupture^39–41^. 18F-NaF positron emission tomography-computed tomography is currently the only imaging modality that can detect atherosclerotic microcalcification;^41^ however, its application in coronary beds is limited due to its poor spatial resolution and presence of cardiac motion artifacts. In the present μOCT study, plaque microcalcification was visualized as distinct bright spot. OCT bright spot was conventionally considered to represent macropahges^42^, however, a recent human autopsy study showed that bright spots can be generated by other plaque components that cause sharp changes in index of refraction^43^. In this reports, the interfaces between calcium and fibrous tissue, or between calcium and lipid were responsible for 15% of bright spot-positive regions^43^. We thought that the presence of dense calcium within macrophage-filled tissue led to a drastic change in refractive indices; thus yielded bright signals in μOCT image. The limited spatial resolution of conventional OCT may not be adequate for imaging this minute change that arise at the interface of tiny calcium.

## Discussion

The introduction of intravascular OCT, an optical imaging technique providing 10-fold higher resolution than intravascular ultrasound, has further increased our understanding of coronary atherosclerosis during the last decade. In the present study, we demonstrated the feasibility of a flexible endoscopic µOCT system for intravascular imaging by testing it in coronary-sized vessels of various animal models, including a healthy swine coronary artery, BVS-implanted swine coronary artery, and rabbit artery with high-risk atherosclerotic plaques. Compared with the conventional OCT, the present technique could provide additional, cellular- or subcellular-level information relevant to atherogenesis, plaque destabilization, stent-artery interaction, and neointimal healing.

To build a clinically translatable intravascular µOCT imaging system, fabrication of miniaturized imaging probe with a helical scanning capability is warranted. It was previously reported that an endoscopic µOCT system was feasible for imaging the swine trachea *in vivo*^44^, however, the rigid endoscope with an outer diameter of 4 mm limited its broader applications. Cui D *et al*. reported the fabrication of a flexible endobronchial µOCT probe with a diameter of 2.4 mm, incorporating a GRIN lens and an apodizing beam splitter to extend DOF^45^. However, tomographic imaging of the entire lumen could not be performed with this endoscopic µOCT system that provided one-dimensional mapping only. Yuan W *et al*. developed an endoscopic µOCT imaging probe with a diameter of less than 1 mm and demonstrated its feasibility for visualizing the luminal organs of animal models^17^. Their imaging probe based on fiber-optic ball lens provided an axial resolution of 2.4 µm; but a sufficient level of transverse resolution (6 µm) was not achieved^17^. This mismatch may considerably hamper the detailed visualization of cellular or subcellular structures. More recently, Yin B *et al*. reported a µOCT system that innovatively extended DOF by implementing few-mode interferometry^46,47^. Their approach based on a small-diameter, flexible catheter successfully visualized cellular features of diseased human coronary arteries and coronary-sized arteries of living rabbits^46^. However, since each mode had a different NA, the lateral resolution created by each mode unavoidably varied, resulting in a non-uniform lateral resolution across depth (average lateral resolution of 3–4 μm)^46,47^. The present µOCT imaging system provided a cellular-level resolutions that were at least equivalent or superior to those reported previously. Moreover, our highly flexible, miniaturized imaging probe had a catheter profile equivalent to that of early intravascular ultrasound. We expect that this highly-translatable intravascular µOCT system allowing visualization of luminal microstructures at an unprecedented level of detail and sensitivity will provide a novel opportunity for the understanding of vascular biology and pathology.

## Methods

### Endoscopic µOCT system

The developed system is based on spectral-domain OCT. A supercontinuum laser (SC400-4, Fianium, Southampton, UK) was used as a light source, transmitting a center wavelength of 850 nm and a bandwidth to 280 nm through a hot mirror (M254H45, Thorlabs, Newton, NJ) and a short pass filter (FESH1000, Thorlabs). We confirmed that the light source was limited to the designed wavelength range using an optical spectrum analyzer. The light goes into an interferometer, which was constructed using a 5: 5 optical fiber coupler (TW850R5A2, Thorlabs) and polarization controller (FPC020, Thorlabs) through a coupling lens (AC050–008-B, Thorlabs). It was confirmed that the coupling efficiency was more than 70% at all wavelength bands used, through ZEMAX simulation. The scanning component was constructed using a lab-built rotary joint, motorized stage (A-LSQ075A, Zaber Technologies Inc., Vancouver, British Colombia, Canada) and the detection part was composed of a custom-designed spectrometer (2048 pixels, Wasatch Photonics, Morrisville, NC); the data were transferred to a computer. The signal obtained by the spectrometer was displayed in real-time as depth information and the processed image and saved on a computer. To acquire three-dimensional images, we developed a lab-built rotary joint for the µOCT system. The rotary joint consisted of two collimators that utilize the GRIN lens (SLW180023083X30, Go!Foton, Somerset, NJ), a rotating motor (3654K024B, Faulhaber, Schönaich, Germany), and a timing belt. One of the collimators was rotated for scanning through the timing belt which transmits the rotation force from the motor. The completed rotary joint was connected to the motorized pullback stage through a stage adapter for helical scanning of the imaging probe.

A schematic diagram of the imaging probe is shown in Fig. 3a. It was composed of a GRIN lens (X_W10-S0180–083-SOC X** =** 4DP, Go!Foton), glass spacer (FG550UEC, Thorlabs), single-mode fiber (750HP, Thorlabs) and gold-coated mirror. First, the single-mode fiber was fusion spliced to the glass spacer, and then precisely polished to a predefined length by polishing machine. The polished surface was angled at 4° to reduce noise caused by back-reflection. Second, the binary phase spatial filter (BPSF) applied GRIN lens and glass spacer were precisely monitored and aligned using a three-axis linear stage, and then bonded using UV curable epoxy. The BPSF pattern has annular patterns, and was fabricated using soft lithography techniques. The details of the fabrication methods and performance of the BPSF are described in our previous publications^21,22^. Third, the imaging probe from the previous step was inserted in to a steel tube with an outer diameter of 1.21 mm, and the gold-coated mirror with an angle of 41° was inserted in the other side of the steel tube. The gold-coated mirror was aligned to allow light to propagate through the window of the steel tube. Thereafter, the entire fiber was encased in a torque coil (Asahi Intecc Co, Aichi, Japan) with outer/inner diameter of 0.65/0.27 mm for protection and transfer of torque for probe rotation and translation. 3D PSF of the imaging probe was measured using a 20x objective lens (#38–330, Edmund optics, Barrington, NJ) and a beam profiler (BC106N-VIS/M, Thorlabs). The objective lens and beam profiler were aligned using a three-axis linear translation stage. We analyzed the PFS at the focal plane defined as the axial position with the highest peak intensity of the measured PSF. The lateral resolution was defined as FWHM of the intensity distribution, which was determined by linear interpolation. When calculating lateral resolution, the side lobes were not considered due to their low intensity (81.8% lower than the main lobe). The axial profile was measured using a mirror (PF10–03-P01, Thorlabs) at the focal plane and axial resolution analyzed as FWHM of linearly interpolated data.

### *Ex vivo* imaging protocol

Imaging was performed twice in each blood vessel at a 10k A-line rate with a rotation speed of 120 rpm and a pullback speed of 50 μm/s and 500 μm/sec to achieve an interval between frames was 25 μm and 250 μm. Each blood vessel was also imaged using a conventional OCT system at the same position with the same frame interval (51.2k A-line rate with a rotation speed of 3000 rpm and pullback speed of 1.25 mm/s and 12.5 mm/s) to allow direct comparison against the µOCT. Intravascular OCT system, used in this study, was built based on a prototype device from a commercial OCT manufacturer (NinePoint Medical, Cambridge, MA, USA). The axial and lateral resolution, defined as FWHM of peak intensity at the focal plane, of the OCT system was 11.58 μm and 22.67 μm, respectively. Following imaging, the animal blood vessels were resected, fixed, and processed for histopathological analysis. All animal studies were approved by the Institutional Animal Care and Use Committee of Korea University College of Medicine (KOREA-2016-0170-C2 and KOREA-2018-0066), and all animal experiments procedures were performed in accordance with the relevant guidelines and regulations.

### Swine model of BVS-implanted coronary artery

To investigate the early arterial healing process following intravascular stent implantation, a swine coronary stenting model was developed. Given that stent healing takes a month to complete in swine^48^, we implanted BVSs twice, with a 3-week interval, and euthanized the animal at fourth week to investigate one BVS in the very early phase (7 days post-implantation) and the other in the final phase (28 days post-implantation) of stent healing simultaneously. The implanted BVSs were poly-L-lactic acid-based polymeric stent (BRS, Suntech, Korea) with strut thickness of 100 μm and outer diameter of 2.5 mm. In the first procedure, we implanted two overlapping BVSs in the left anterior descending artery. *Ex vivo* imagings were performed using a custom-made imaging chamber (Supplementary Fig. S2), as previously described^49^.

### Rabbit model of atherosclerotic microcalcification

Rabbit model of atherosclerotic microcalcification was developed using previous methods with some modifications^50^. New Zealand white rabbits (male sex, 2.5–3.0 kg, DooYeol Biotech, Korea) were fed a high cholesterol diet containing vitamin K1 (1% cholesterol + 1.5 mg/g of vitamin K1; DooYeol Biotech) for 4 weeks and then with a high cholesterol-warfarin diet (1% cholesterol + 1.5 mg/g of vitamin K1 + 3 mg/g of warfarin; DooYeol Biotech), which was maintained for an additional 8 weeks.

### Histologic validation

After μOCT imaging, the frozen swine coronary arteries were serially sectioned and stained. PM-2K monoclonal antibody (Abcam, Cambridge, UK) was used for the identification of plaque macrophages and α-SMA antibody (Abcam, Cambridge, UK) for SMCs. We used Modified VMT staining technique to highlight elastic fibers and other connective tissue elements. The rabbit arteries were fixed in 10% formalin and processed into paraffin-embedded blocks. Four-micrometer paraffin sections were prepared using a Leica RM2255 microtome (Leica Biosystems). The sections were deparaffinized and rehydrated, and then H&E and von Kossa (CVK-2-IFU, ScyTek Laboratories, West Logan, UT) staining were performed in accordance with the manufacturer’s protocol. For immunohistochemical analysis, antigen retrieval was performed, endogenous peroxidase was blocked; and plaque macrophages were stained using RAM11 primary antibody (M0633, Agilent Dako, Santa Clara, CA). Tissue sections were then labelled with the Envision Polymer Detection System (Agilent Dako).

## Supplementary information

Supplementary Movie 1

Supplementary Materials.
