# Supplementary material for: Flexible endoscopic micro-optical coherence tomography for three-dimensional imaging of the arterial microstructure
Source: Sci Rep. 2020 Jun 8;10:9248. doi: 10.1038/s41598-020-65742-2 (PMC7280224; doi:10.1038/s41598-020-65742-2)
Supplement: Supplementary file 2 — Supplementary Materials. [file 41598_2020_65742_MOESM2_ESM.docx]

**Supplementary Materials**

**Flexible endoscopic micro-optical coherence tomography for three-dimensional imaging of the arterial microstructure**

Junyoung Kim^1, +^, Sunwon Kim^2,3 +^, Joon Woo Song^3, +^, Hyun Jung Kim^3^, Min Woo Lee^4^, Jeongmoo Han^5^, Jin Won Kim^3, *^, and Hongki Yoo^5, *^

^1^Mechanical Engineering Research Institute, KAIST, Daejeon, 34141, Republic of Korea

^2^Department of Cardiology, Korea University Ansan Hospital, Ansan, 15355, Republic of Korea

^3^Multimodal Imaging and Theragnostic Laboratory, Cardiovascular Center, Korea University Guro Hospital, Seoul, 08308, Republic of Korea

^4^Center for Robotics Research, Korea Institute of Science and Technology, Seoul, 02792 , Republic of Korea

^5^Department of Mechanical Engineering, KAIST, Daejeon, 34141, Republic of Korea

[^*^kjwmm@korea.ac.kr](mailto:*kjwmm@korea.ac.kr), [^*^h.yoo@kaist.ac.kr](mailto:*h.yoo@kaist.ac.kr)

Non-uniform rotational distortion analysis


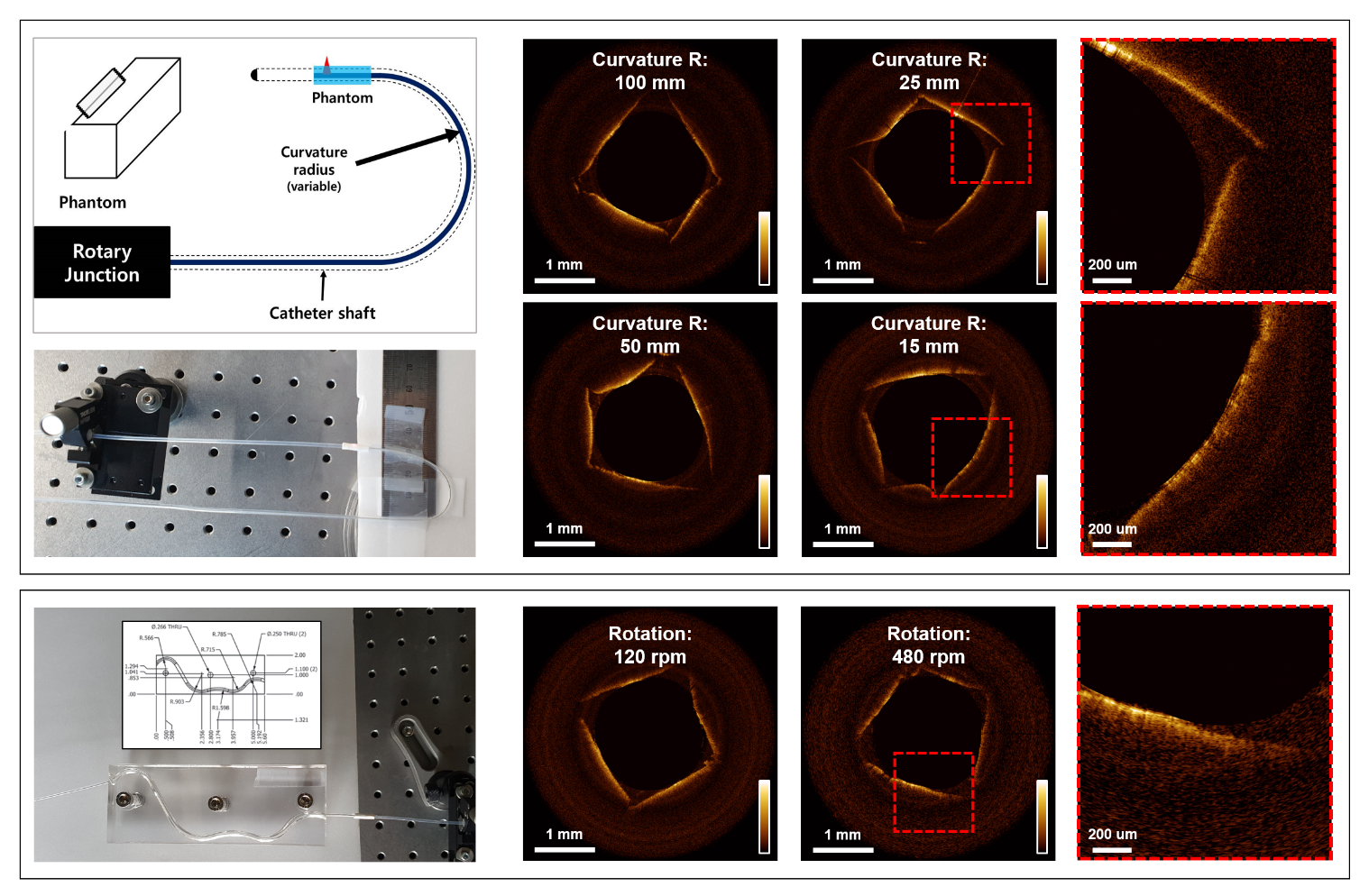
Non-uniform rotational distortion (NURD) may occur as a result of rotational friction between the torque coil and hollow drive-shaft when the catheter is introduced into a tortuous anatomy via a curved guiding catheter. To investigate whether the current µOCT catheter can be applied to tortuous arterial environment, we conducted phantom imaging experiments with the catheter shaft being held bent differently (Supplementary Fig. S1, upper panel). OCT images were acquired with a rotation speed 120 rpm at each curvature radius ranging from 100 mm to 15 mm. Also, imaging was also acquired with the catheter shaft being placed in five-point-curved tortuous vascular phantom at rotation speed range of 120 rpm to 480 rpm (Supplementary Fig. S1, lower panel). Our flexible imaging catheter did not produce NURD under any of the above mentioned conditions.

Supplementary Fig. S1. Upper panel, Left, Schematic illustration and actual picture showing phantom imaging experiment. Right, A 180-degree bent catheter provides NURD-free µOCT images at varying curvature radii. Lower panel, Imaging experiment using a tortuous vascular phantom. The last column shows high-magnification images.


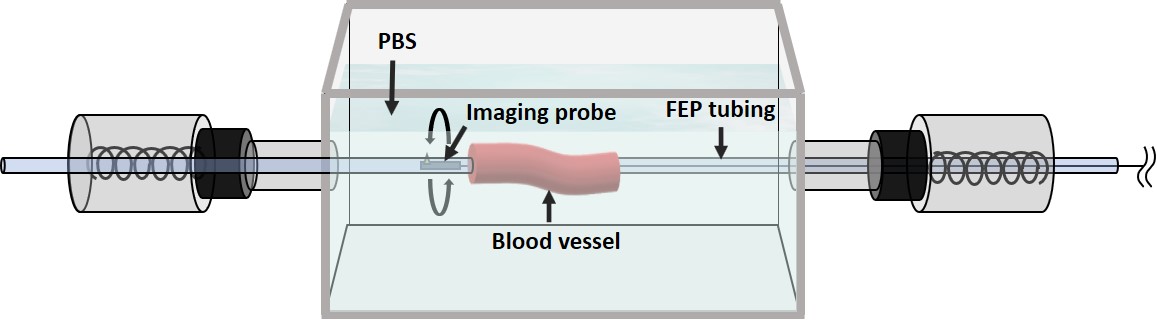


Supplementary Fig. 2 Illustration of the custom-made *ex vivo* imaging chamber


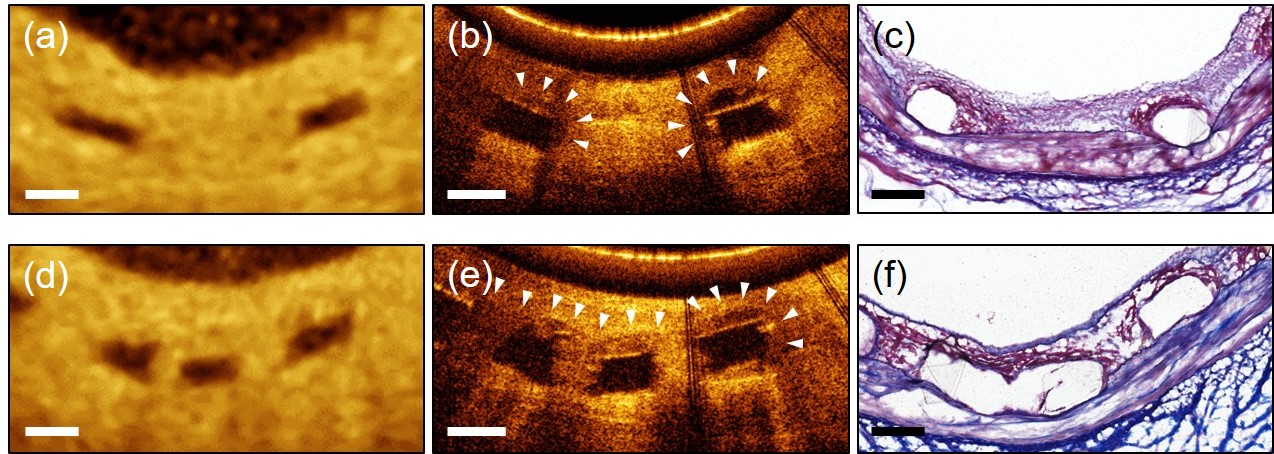


Supplementary Fig. 3 Imaging results of a BVS-implanted swine coronary artery (28 days post-implantation). a, d Conventional OCT cross-section images; b, e μOCT images showing small peri-strut low intensity areas (white arrowheads); c, f Corresponding histologic sections demonstrates the presence of mild fibrin deposition around struts (Verhoeff elastic-Masson trichrome stain). Scale bars, 200 μm.

Supplementary Table 1 Technical specifications of the conventional OCT and µOCT that were used in this study.

|  | **Conventional OCT** | **µOCT** |
| --- | --- | --- |
| **Technical Characteristics** |  | |
| Axial resolution | 11.58 μm | 1.83 μm |
| Lateral resolution | 22.67 μm | 3.38 μm |
| Depth of field | 1.31 mm | 281.30 um |
| Maximum A-line rates | 51.2 kHz | 40.0 kHz |
| Imaging probe diameter / outer sheath diameter | 0.60mm / 0.84 mm | 1.20 mm / 1.65 mm |
| Scanning method | Proximal scanning | Proximal scanning |

OCT = optical coherence tomography

**Supplementary Movie 1.** Movie of μOCT imaging obtained from a BVS-implanted swine coronary artery.

Movie file is provided online
